# When Cyclodextrins Met Data Science: Unveiling Their Pharmaceutical Applications through Network Science and Text-Mining

**DOI:** 10.3390/pharmaceutics13081297

**Published:** 2021-08-19

**Authors:** Juliana Rincón-López, Yara C. Almanza-Arjona, Alejandro P. Riascos, Yareli Rojas-Aguirre

**Affiliations:** 1 Instituto de Investigaciones en Materiales, Universidad Nacional Autónoma de México, Ciudad Universitaria, Mexico City 04510, Mexico; juliana.rincon@comunidad.unam.mx; 2 Instituto de Ciencias Aplicadas y Tecnología, Universidad Nacional Autónoma de México, Ciudad Universitaria, Mexico City 04510, Mexico; yara.almanza@icat.unam.mx; 3 Instituto de Física, Universidad Nacional Autónoma de México, Ciudad Universitaria, Mexico City 04510, Mexico

**Keywords:** cyclodextrin, patents, network science, text-mining, dosage forms

## Abstract

We present a data-driven approach to unveil the pharmaceutical technologies of cyclodextrins (CDs) by analyzing a dataset of CD pharmaceutical patents. First, we implemented network science techniques to represent CD patents as a single structure and provide a framework for unsupervised detection of keywords in the patent dataset. Guided by those keywords, we further mined the dataset to examine the patenting trends according to CD-based dosage forms. CD patents formed complex networks, evidencing the supremacy of CDs for solubility enhancement and how this has triggered cutting-edge applications based on or beyond the solubility improvement. The networks exposed the significance of CDs to formulate aqueous solutions, tablets, and powders. Additionally, they highlighted the role of CDs in formulations of anti-inflammatory drugs, cancer therapies, and antiviral strategies. Text-mining showed that the trends in CDs for aqueous solutions, tablets, and powders are going upward. Gels seem to be promising, while patches and fibers are emerging. Cyclodextrins’ potential in suspensions and emulsions is yet to be recognized and can become an opportunity area. This is the first unsupervised/supervised data-mining approach aimed at depicting a landscape of CDs to identify trending and emerging technologies and uncover opportunity areas in CD pharmaceutical research.

## 1. Introduction

Cyclodextrins (CDs) are outstanding materials in the pharmaceutical field, where they have mainly performed as molecular containers of hydrophobic drugs for olubility enhancement. Likewise, through the apparent modification of guest molecules’ physicochemical properties, CDs can improve the stability and organoleptic properties of a pharmaceutical formulation, compelling their use in developing dosage forms for their administration for practically any route [1,2]. Furthermore, CD derivatives are becoming relevant in developing drug-loaded bioadhesive materials [3,4], and CD-based cancer nanomedicines have reached or are currently in clinical trials [5].

More than 40 formulations containing native or modified CDs are currently marketed for diverse therapeutic purposes (Table S1, Supplementary Material). Furthermore, their use is expanding, as evidenced by at least six formulation approvals in the last five years (Table 1), which include Baqsimi^®^ (Eli Lilly and Company, Indianapolis, IN, USA) for the nasal delivery of glucagon and Trappsol^®^Cyclo^TM^ (Cyclo Therapeutics, Inc., Gainesville, FL, USA), for the treatment of Niemann–Pick disease type C, in which the active pharmaceutical ingredient (API) is the CD itself [6,7]. Two recent breakthroughs are Veklury^®^ (Gilead Sciences, Inc., Foster City, CA, USA) (SBEβCD/remdesivir) to treat hospitalized patients with severe COVID-19 and the Janssen COVID-19 vaccine containing HPβCD, which were FDA-approved for emergency use in 2020 and 2021, respectively [8,9].

Technological interest in CDs within the pharmaceutical field is continuously growing, and monitoring CD-based pharmaceutical technologies is fundamental to identifying emerging technologies and uncovering promising opportunity areas, which ultimately may support decision-making in CD pharmaceutical research.

Technological information, a substantial component of research and development, is commonly found in patents; hence, the analysis of patent documents may provide indicators for novel developments, inform about emerging technological areas, and support the identification of opportunities for technological forecasting [12].

As some technologies and innovations in a patent are not usually published in scientific literature, important information can remain hidden from researchers [13]. Therefore, articles rarely cite patents, although patents often cite articles. Thus, understanding the interplay between scientific literature and patents is a practical approach to prioritize investigations and investment and forecast the success of specific research in academia [12,14,15]. Moreover, it can foster innovations quickly, particularly when the innovation cycle becomes more complex and shorter and when the market demands rapid responses [16]. Such is the case of the efforts to develop therapies to treat or prevent SARS-CoV-2 infection [15,17]. 

The content, length, and structure of patents are different from that of scientific articles. Additionally, patents display an intricated writing style, characterized by complex and long sentences that could shield important information. This particular communication method can make the analysis of patents a challenging task [18].

Network science studies emergent patterns in a system, considering their parts and interactions [19]. In general terms, a network consists of nodes (vertices) that describe the elements of the system and edges (links) that represent relationships between the elements. Network science is a fundamental paradigm in the description of complex systems, and it has gained enormous importance in the understanding of social networks, citation networks, bioinformatics, and even the functional organization of a living cell [19,20,21,22].

Although little has been done in the analysis of patent data through network science, there is evidence that it is a valuable tool that provides a mathematical framework to analyze patents at different levels, from single documents to complete text databases, and may allow the obtaining of a patent landscape in which patents can be analyzed as a whole while detecting the relationships among them. Community detection algorithms in a network of patents allow the identification of sets of nodes densely connected internally but with reduced connectivity between communities; this particular organization serves as a clustering method for the unsupervised classification of patents with similar content. General clustering techniques constitute an essential data mining component and are fundamental in unsupervised machine learning tasks [23,24]. Thus, community detection in the patent landscape may unveil low-patented technological areas, emerging trends, and even academic or industry partners to collaborate with in future research [12,15,25].

**Figure 1:**
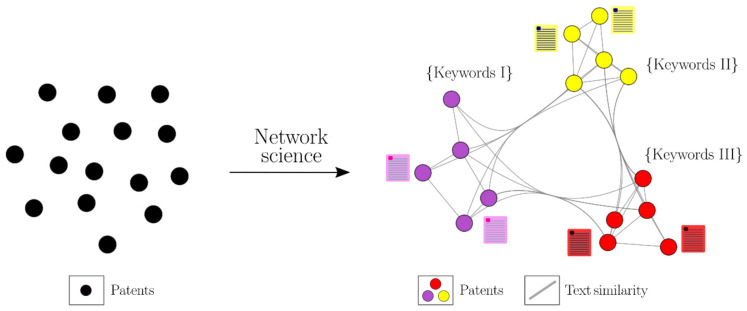
CD patent analysis and keyword identification through network science.

We recently reviewed the evolution of CD pharmaceutical technologies in terms of administration routes by analyzing a dataset of 1998 pharmaceutical CD patents retrieved from the Derwent Innovation Index database [1]. The review was achieved by text-mining techniques that enabled knowledge extraction from patent texts according to their semantic content. This review is the first of its type for CD pharmaceutical applications. Nonetheless, that work required specific sets of keywords, necessarily provided by experts in the field. Motivated by the fascinating unsupervised data-driven approaches to extract knowledge from data, herein, we present a research work divided into two stages. In the first stage, we mine the same dataset, but, this time, by implementing network science tools to establish a coarse-grained unsupervised technological representation that automatically retrieves knowledge in the form of keywords describing groups of patents without prior expertise in this field, thus reducing possible bias in the interpretation of results. In this way, we represented all the CD patent information as a network, where each node represents a patent and connections describe their similarity (Figure 1).

In the second stage, the keywords provided by the networks guide a further text-mining of the patent dataset, the discussion of which was based on the obtained networks and supported by our previous review. The whole analysis was based on the patent’s semantic content in the “*Novelty*” and “*Use*” sections of the document.

The increasing availability of data in different scientific fields conceives significant opportunities to leverage the data to guide research [26,27,28,29,30,31,32,33]. Thus, data-driven approaches based on methods similar to those presented herein [18,34,35], along with recent discoveries in machine learning, natural language processing, and text-mining, can pave the way for scientific discoveries and innovation in the pharmaceutical field for both academia and industry.

## 2. Methods

### 2.1. Analysis of CD Pharmaceutical Patents by Network Science

**Figure 2:**
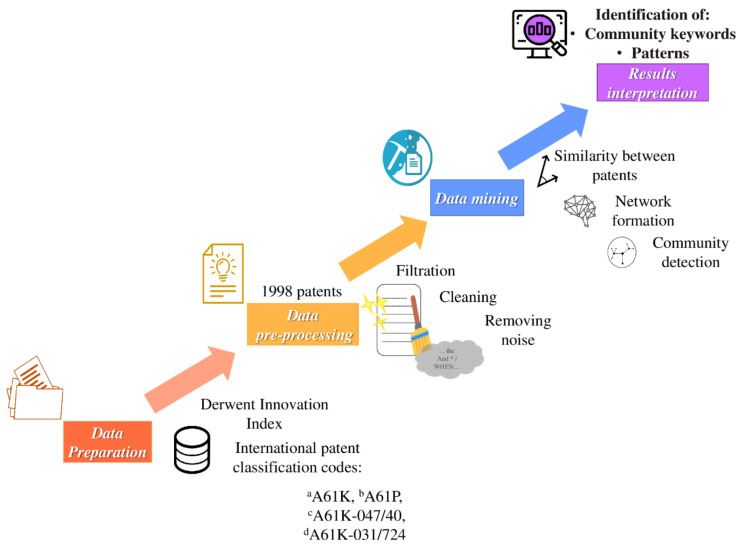
Data mining flowchart. ^a^A61K: preparations for medical, dental, or toilet purposes; ^b^A61P: specific therapeutic activity of chemical compounds or medical preparations; ^c^A61K-047/40: cyclodextrins and derivatives thereof (medicinal preparations characterized by the non-active ingredients); ^d^A61K-031/724: cyclodextrins (medicinal preparations containing active organic ingredients). A61Q, referring to “specific use of cosmetics or similar toilet preparations”, was excluded from our search.

We followed the flowchart shown in Figure 2 to mine the CD pharmaceutical patents according to their semantic content. The description of each one of the steps is given below. 

#### 2.1.1. Data Preparation

We employed the 1998 patent dataset, whose retrieval was previously reported [1]. Briefly, all patents containing the truncated keyword *cyclodextrin* were collected from the Derwent Innovation Index (DII) database (Clarivate, 2020; access through the National Autonomous University of Mexico, Mexico City, Mexico) until 2019. We used specific International Patent Classification codes to delimit the search (Figure 2).

#### 2.1.2. Pre-Processing

We developed a classification method that extracted the information in the fields of “*Novelty*” and “*Use*” in each patent based on the following pre-processing operations:

- Punctuation and symbols. We removed punctuation and special characters, such as numbers and math symbols, and lowered all words.
- Stop words. We removed commonly used stop words (such as “the”, “in”, and “is”) that are unnecessary in patent classification.
- Text lemmatization. In this stage, families of words derived from a unique root were replaced by a unique base or dictionary form known as the lemma.
- Common words. Because we were interested in specific CD applications, we removed those words commonly found in the text that did not provide a particular context. In this category, we included different types of words such as adjectives, verbs, and adverbs, for example, “consists”, “contain”, “effect”, “enables”, “excellent”, “exhibit”, “good”, “main”, “method”, among others.

These pre-processing operations were implemented in Python using the Natural Language ToolKit [36] and pandas [37] libraries.

#### 2.1.3. Data Mining

We created a ranking of words based on their frequency and position in each text from the list of words obtained in the pre-processing stage. After that, we chose the first M-classified words, meaning that M words describe each patent with their frequencies. Using all the pre-processed dataset words, we defined a d-dimensional Euclidean space of keywords in which a patent with M non-null entries, with its respective frequencies of words, is represented as a vector. After that, we defined a measure to quantify the similarity between two patents. 

We used the cosine similarity that provides a measure proportional to the angle between patents i and j; we defined the similarity coefficient $c_{ij}=\frac{2}{\pi }arccos\left[\frac{{\hat{v}}_{i}\cdot {\hat{v}}_{j}}{\left|{\hat{v}}_{i}\right|\left|{\hat{v}}_{j}\right|}\right]$, where $\hat{v_{i}}$ denotes the vector describing the patent i, ${\hat{v}}_{i}\cdot {\hat{v}}_{j}$ is the dot product between vectors, and $|{\hat{v}}_{i}|=\sqrt{{\hat{v}}_{i}\cdot {\hat{v}}_{i}}$ is the norm. Specifically, we have the value $c_{ij}=0$, if the list of selected words representing two patents coincides; this is the case when a patent is compared to itself. When all the words examined in patents i and j are different, we have $c_{ij}=1$.

Afterward, we statistically analyzed the $c_{ij}$ similarity coefficients to define a threshold value H, which determines whether two patents are connected or not. Thus, by using the similarity coefficients and H values, we generated a network of patents in which a link (connection) between two different patents i and j is established if $0<c_{ij}\leq H$, that is, a network described by a matrix A (denoted as $A_{ij}$), with element 1 if two nodes (patents) are connected and element 0 otherwise; therefore, $A_{ij}=1$ if $0\leq c_{ij}\leq H$ and $A_{ij}=0$ for $c_{ij}>H$ for our network. By definition, the matrix considers the diagonal entries $A_{ii}=0$ to avoid loops or connections of a node to itself.

Once the structure was defined, we applied different Python library tools [38] for its analysis. We centered our analysis on the largest connected component (LCC), which detects the largest set of connected nodes within the network; this subnetwork does not include individual or small patent clusters. On the other hand, we studied network degrees, defined as $k_{i}=\sum_{l=1}^{N}A_{il}$ (N being the size of the LCC), that provided the number of connections of the patent i. The statistical analysis of those degrees allowed us to define the network type [21].

Finally, we applied the Louvain algorithm [39] to detect communities in the network; these are groups of patents in which connections between nodes are denser than connections among the rest of the network [19]. In other words, we detected groups of patents with similar semantic content. 

The analyses were conducted in Python, and the codes are available from the authors upon request.

#### 2.1.4. Interpretation

Community detection allowed us to further expose the relative frequency of words in each one of the groups. Then, by analyzing the ten most common words found for each group, we identified predominant patterns in the organization of words within the CD patent landscape. 

### 2.2. Text Mining

The resulting network showed the presence of keywords associated with dosage forms in most of the communities. Therefore, we carried out further mining of the 1998 patent dataset, guided by words corresponding to pharmaceutical dosage forms, aiming to determine the number of patents related to each of them and observe how they have evolved. We employed our previously reported techniques to identify specific words in each patent [1]. The words used in the queries for dosage forms were based on U.S. Pharmacopeia definitions [40].

## 3. Results and Discussion

### 3.1. Data Mining by Network Science

The pre-processing stage resulted in a subset of data containing those terms providing the context to detect groups of patents containing similar keywords. Considering M=5 keywords, we found 2807 and 2223 words describing the patents in *Novelty* and *Use*. These words also defined the dimension of the Euclidean space for both fields. At this stage, the patents were represented as points distributed in that space, in which the distance between them (cosine similarity) can be measured, something that would not be possible by just comparing the texts and reading them.

**Figure 3:**
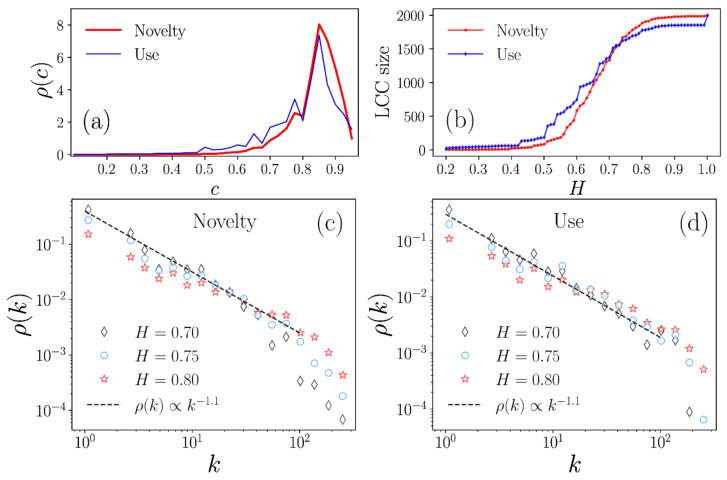
Characterization of similarity networks from the semantic content analysis in the *Novelty* and *Use* sections of CD patents. (**a**) Statistical analysis of similarity values c in the interval 0.1≤c≤0.95; (**b**) LCC size generated with threshold values H; probability density of the k degrees for the networks generated for H=0.7, 0.75, 0.8 within the (**c**) *Novelty* and (**d**) *Use* fields.

We computed the cosine similarity coefficients $c=c_{ij}$ between all patents i,j in these spaces, storing the information in an N×N matrix, with N=1998 (patents in the dataset). Afterward, through a statistical analysis of all c values, we determined the probability density $\rho \left(c\right)$ for the words in the *Novelty* and *Use* fields (Figure 3a). In both cases, the curves showed a relative maximum around c=0.85, and a high fraction of the similarity values were in the interval of  0.6<c<1. 

Similarity coefficients provided the necessary information to build a network of patents: a multidimensional representation for global data analysis, in which each node is a patent, and links or connections are defined in terms of the threshold value H between two patents. We identified a range of c to establish the parameters for constructing the patent network. Then, we explored different threshold H values to determine if two i, j patents are connected if $0<c_{ij}\leq H$, considering that for small H values, only highly similar patents are joined, while when H is close to 1, nested structures with minor restrictions are produced, and a higher number of patents are connected. On the other hand, H=1 depicts a fully connected network in which a link would connect all patents. 

The effect of H on the network was determined based on the LCC that indicates the number of nodes of the largest connected network, obtained through a given threshold value H. As seen in Figure 3b, H<0.5 generates LCCs that include only a few patents, whereas values of  H≥0.7 produce connected networks that include a high fraction of the 1998 patents.

In that way, we analyzed the structure of networks with threshold values H=0.7, 0.75, 0.8. For each one of them, we calculated the degree k, which denotes the number of connections of a patent. The subsequent statistical analysis [21] informed us that the probability density of the degree $\rho \left(k\right)$ follows an inverse power-law $\rho \left(k\right)\propto k^{-\gamma }$, a behavior observed for our dataset patents with 1≤k≤100  connections (Figure 3c,d illustrates the case $\rho \left(k\right)\propto k^{-1.1}$, with dashed lines as a guide). In network science, structures with a power-law distribution $\rho \left(k\right)\propto k^{-\gamma }$ are called scale-free and describe a hierarchical structure in which their nodes, arranged in small groups, organize hierarchically in increasingly larger groups through links connecting the whole network, namely, a complex network [19,41]. Therefore, our findings revealed that the CD pharmaceutical patents, when analyzed through semantic content similarity in terms of their *Novelty* and *Use* fields, are organized in complex networks. This fascinating behavior has been observed in different systems, such as biological, social, and communications [19,41].

#### 3.1.1. Community Detection in CD Patents’ Complex Networks

Once we generated the networks with different *H* values, we aimed to detect communities by applying the Louvain algorithm. Community detection endorses identifying local patterns and guides the understanding of community interaction in a complex structure. In this work, the communities represent groups of patents with similar semantic content, which arose from considering all the information contained in the network, something not immediately visible if comparing the patent texts in a standard table or chart.

**Figure 4:**
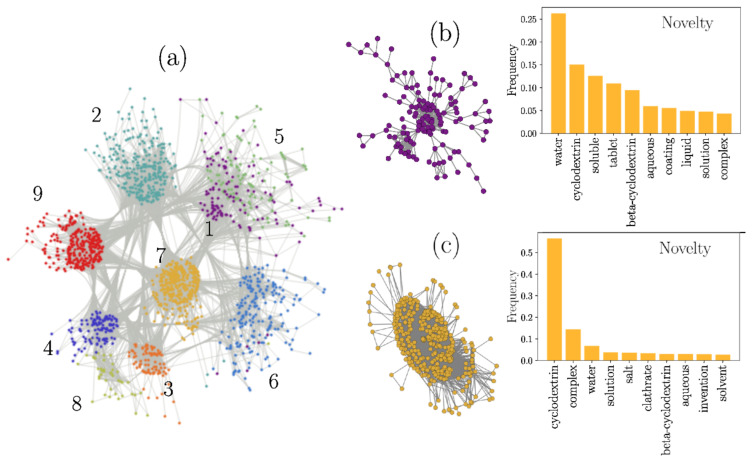
Graphical representation of the complex network formed from CD patents for the *Novelty* field. (**a**). Communities 1 (**b**) and 7 (**c**), with their histograms representing the relative frequency of the 10 most common words found for each community.

For the case of the *Novelty* field, the network was generated with a threshold value H=0.75. With this choice, the LCC is constituted by N=1623 nodes, organized in nine communities (shown in different colors in Figure 4a). In this representation, we see how communities are formed by subsets of nodes that are intensely connected to each other. To exemplify the communities’ topology, we selected Communities 1 and 7, depicted in Figure 4b, accompanied by their respective histograms, showing the relative frequency of the 10 most common words found for each community.

**Figure 5:**
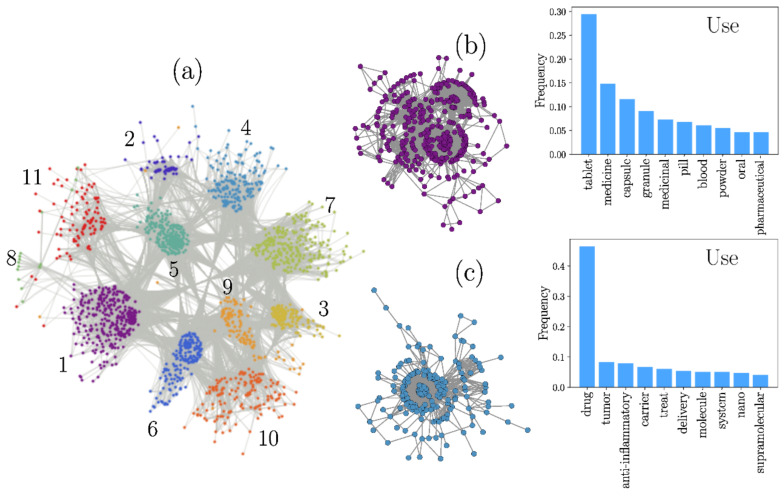
Graphical representation of the complex network formed from CD patents for the *Use* field (**a**); Communities (**b**) 1 and (**c**) 4, with the histograms representing the relative frequency of their 10 most common words.

A similar analysis was carried out for those words analyzed in the *Use* field. In this case, through exploring different configurations, we selected the threshold value of H=0.8 to define the similarity structure with an LCC of N=1779. This larger H value makes the similarity of patents less restrictive than in the *Novelty* network. Here, the Louvain algorithm detected 11 clusters (Figure 5a). Figure 5b depicts Community 1 and Community 4 as examples to observe their topology. The relative frequency of their 10 most common words is also included.

#### 3.1.2. Analysis of Community Keywords

Comparing the semantic content for all pairs of patents of our dataset, achieved by implementing the approaches described above, was the primary motivation of this work. In this way, network science provided a natural conceptual framework for a multiscale description of the patents’ similarity. On a large scale, we found a complex network encompassing the semantic similarity of patents as a whole. At the intermediate scale, it was possible to visualize patents clustering into groups containing information that, at the finest scale, formally partitioned the dataset in order to identify patterns through the analysis of those keywords that describe the semantic content of the patents in each community. Table 2 shows the five most common words and their respective relative frequencies, associated with each community, for the *Novelty* and *Use* networks.

#### 3.1.3. Novelty

The words ranking revealed that CD pharmaceutical patents’ *Novelty* relies primarily on incrementing drug solubility to formulate aqueous solutions. This is evident when observing Community 7, the largest in the network. Within the words comprising this community, we found *complex*, *water*, and *solution*, which refers to CDs’ use to increase the water solubility of a drug by forming inclusion complexes, facilitating their formulation as aqueous solutions. This cluster confirms the supremacy of CDs as solubilizers in the pharmaceutical field. 

The same is true for Community 4 and Community 1. The latter contains the word *tablet*, pointing out CDs’ importance in these dosage forms [42]. Community 6, also significant in size, includes the word *powder*, which might refer to a final dosage form (solutes for reconstitution) or to intermediates that are further processed to produce other formulations. A broader discussion about CD patents for tablets, powders, and other dosage forms is presented in Section 3.2.

Community 8 shows the words *oil* and *volatile*, bringing to light the role that CDs have played in facilitating the incorporation of these compounds into suitable formulations, either as amorphous powders or by overcoming the low water solubility and instability associated with them [43,44].

To summarize, it is observed that the *Novelty* of pharmaceutical patents of CDs consists of the aqueous solubility enhancement of drugs for their proper formulation as aqueous solutions. Additionally, the importance of tablets and powders was revealed.

#### 3.1.4. Use

In this network, the patents are grouped according to their pharmaceutical forms, which might be expected since the ultimate goal of using CDs is to develop optimal dosage forms by improving drug solubility, stability, and permeability, among other properties. Nonetheless, target diseases and drug families also made the patents form communities.

#### 3.1.5. Dosage Forms

Community 1, the largest of the network, is mainly associated with tablets, in agreement with Community 1 for *Novelty*, confirming the relevance of CDs or CD/drug ICs in those dosage forms. 

Community 11, although small, confirms that CDs are advantageous in formulations to treat eye disorders. The use of CDs to develop eye drops and other ophthalmic ailments, including CD-based macular degeneration therapies, has gained significant importance over time [45], with an increasing number of patents observed in the last ten years [1]. The following are two examples of recent patents in the field: (1) A formulation of an aqueous ophthalmic solution of brinzolamide to treat ocular hypertension and open-angle glaucoma using HPβCD as a solubilizer agent [46]; (2) a formulation of at least two of the following active agents—a corticosteroid, a non-steroidal anti-inflammatory drug (NSAID), and an antibiotic—to treat a variety of eye conditions. By forming CD/NSAID and CD/antibiotic ICs, soluble and stable formulations were obtained [47]. Fascinating research in the field indeed announces that this upward trend will continue [48,49,50,51,52].

Community 11 also contains the word *nasal*. CDs’ abilities to enhance drug solubility, permeability and optimize organoleptic properties make them attractive components for nasal formulations. Proof of this is a recent patent describing an intranasal epinephrine formulation to treat medical emergency hypersensitivity reactions, such as anaphylaxis, in an out-of-hospital setting, in which CDs work as absorption enhancers of the drug [53]. Another striking example, in which the CD is the active ingredient, is an aqueous solution containing 2,6-di-O-methyl-βCD (DIMEB), which, after nasal administration, is effective in treating prion disease. This patent showed how the intranasal administration of 0.032 mg/day of DIMEB significantly increased the survival of C57BL mice suffering from the disease [54]. Hence, the increasing significance in nasal delivery and compelling investigations on nasal mucoadhesive CD-based materials could soon make these technologies gain more strength [55,56].

#### 3.1.6. CDs in Cancer

Communities 3 and 4 highlight the interest in using CDs for cancer therapies. Several anticancer drugs are characterized by low solubility, poor intestinal permeability, and low bioavailability, hampering the development of suitable formulations for parenteral or enteral administration. CDs have been widely investigated to overcome these drawbacks, either as CD/chemotherapeutic non-covalent ICs or through CD-covalent conjugates as chemotherapeutic delivery platforms [57]. Some CD-based anticancer treatments are currently under clinical evaluation [5]. Several patents concerning CDs and cancer therapies were found within our dataset. We chronologically describe some examples below.

A patent filed in the late 1980s disclosed the solubility enhancement of 1,1-cyclobutanedicarboxylatediammineplatinum (II) (a cis-platinum derivative compound) through the complexation with αCD to allow the preparation of solutions for parenteral or oral administration [58]. 

The interest in patenting CD/chemotherapeutic ICs continued during the 1990s. For instance, the CD/toremifene IC enabled the formulation of topical preparations to treat cancers localized in the skin or a short distance from the skin, such as metastatic lesions of breast cancer [59]. In another case, the complex DMβCD/taxol increases the apparent taxol solubility to facilitate its absorption when administered either through the IV or oral route to cancer patients [60].

SuperGen, Inc. (now Astex Pharmaceuticals, Inc., Pleasanton, CA, USA) presented an exciting application of CDs, beyond drug solubility enhancement, addressing the reduction of vascular irritation and extravasation when a combination of CDs and antineoplastic drugs were administered intravenously [61].

In recent years, the exhaustive investigation of CD-based chemotherapies in the nanomedicine field has brought about several promising approaches. One of them is CRLX101, a CD-based nanoparticle consisting of camptothecin conjugated to a copolymer of βCD and polyethylene glycol that achieves the sustained release of the drug with high systemic concentration. This nanosystem is now in Phase I/II clinical trials for small cell lung cancer, with a completion date estimated at 2027 [62,63,64]. The success of CRLX101 prompted similar CD-based polymeric nanotechnologies, also patented by Cerulean Pharma Inc., for example, the CD–camptothecin conjugate combined with bevacizumab for advanced renal carcinoma [65,66] and linear CD polymers, covalently bound to a therapeutic agent such as etoposide or tubulysin, for the treatment of breast, lung, colon, and ovarian cancer. For the latter example, efficacy studies in implanted HT-29 colorectal carcinoma xenografts, treated with the CD polymer tubulysin, resulted in substantial antitumor activity during a 90-day study, without significant toxicity [67].

In 2019, Yale University patented spatiotemporally tuned particles (STPs) for spatial and temporal delivery of two or more agents to the same targeted cell. The STPs comprise a polymeric core containing one drug and a functional surface that could be a CD for further complexation with another drug, the first to be released. In vivo studies on female C57BL6/J mice administered with SPTs and tamoxifen demonstrated a superior immune tolerance compared to simple co-encapsulated nanoparticles [68]. Undoubtedly, CDs have accompanied the search and development of cancer therapies, in which they have been used as solubilizers, and, more recently, in the development of platforms for the controlled release of anticancer drugs. These efforts are expected to bear fruit in the short term.

#### 3.1.7. CDs in Antiviral Therapies

The case of Community 10 reflects the appealing role that CDs have played in developing antiviral therapies. Several antivirals are far ideal due to their low aqueous solubility, low permeability, and short half-life. CDs can form ICs with some of these drugs, overcoming the limitations mentioned above [69,70]. Such is the case of remdesivir, the first recommended drug to treat COVID-19, a low water-soluble compound formulated as an IC with SBEβCD for IV administration [71]. CDs have also been explored in conjugates [72] and nano delivery systems [73]. CDs also exhibit an antiviral profile by themselves [74,75]. Moreover, HPβCD has debuted in the field of vaccines, with its use in the Janssen COVID-19 biologic [8,76]. The following are some examples of patents employing CDs in the development of antiviral therapies. 

A patent registered in the early 1990s disclosed a preparation for intranasal administration to treat the common cold caused by rhinoviruses of ICs formed from CDs and diverse antiviral compounds. The preparation enabled continuous and controlled drug delivery for sustained periods [77]. An invention filed in 2003 described pharmaceutical compositions of antiviral proteins (i.e., cyanovirins) for the treatment or prevention of infections caused by retroviruses, in particular HIV-1 or HIV-2, in which CDs perform as absorption enhancers [78]. In another case, βCD was used to block viruses’ ability to infect the cells by disrupting the host’s lipid raft structure through cholesterol extraction to treat or prevent AIDS, genital herpes, or human papillomaviruses [79]. Another innovation describes the use of 3,3′-di-indolymethane to treat respiratory syncytial virus infection and how CDs enable a diversity of formulations: aerosols, dry particles for oral use, and emulsions for IV and parenteral administration. The formulations resulted in a significant reduction in viral counts in the lung tissues of RSV-infected Balb/c mice [80]. A recent patent claims a CD derivative as a broad spectrum virucidal. The CD is functionalized with sulfonic acid groups to mimic the negatively charged surface of cell receptors, commonly used by the viruses for attachment (i.e., heparan sulfate proteoglycans), thus preventing virus entry into cells [81]. Certainly, CDs’ versatility in the development of antiviral therapies makes them an appealing tool that is gaining attention in the fight against viral infections, including SARS-CoV-2 [70,82,83].

#### 3.1.8. CDs and Anti-Inflammatory Drugs

Optimization of formulations containing anti-inflammatory drugs has succeeded with several commercial products (Table S1). This could explain Community 5, one of the largest within the network generated under the “*Use*” context. The same is true for Community 7, pointing out CDs to formulate analgesics through the word *pain*. As expected, numerous patents have been published on this topic over the last decades. For example, in 1992, Chiesi Farmaceutici, S.p.A. filed a patent related to preparing βCD/piroxicam ICs and their different dosage forms. When formulated as tablets, they resulted in a higher dissolution rate than piroxicam alone, improving pharmacokinetics and gastric tolerability. Additionally, the ICs were advantageous to the preparation of pharmaceutical compositions for rectal and topical administration [84]. βCD/piroxicam oral tablets are still marketed in Europe by the same laboratory under the trade name of Cycladol^®^ (Chiesi Farmaceutici S.p.A., Parma, Italy). 

In another example, a CD/ibuprofen clathrate to be taken as a hot drink was patented in 1993 [85]. Other recent patents describe CD/NSAID aqueous solutions or liquid throat sprays [86,87]. Interestingly, Albuquerque et al. published a patent related to the preparation of CD conjugates with anti-inflammatory drugs, displaying anti-tumoral properties, exemplifying Community 4, which contains both words [88]. Comprehensive reviews about patents of CDs and anti-inflammatory drugs and analgesics have been presented before [89,90].

In summary, the analysis of both *Novelty* and *Use* complex networks reaffirmed how CDs have supported the preparation of aqueous solutions by enhancing drug solubility and exposed the relevance of CDs in tablets and powders. Additionally, it pointed out the important role that CDs have played in the formulation of anti-inflammatory drugs, the optimization of cancer therapies, and the development of antiviral strategies. 

It is well known that a complex network is the consequence of a growth process (the increment in the number of nodes over time) with preferential attachment, meaning that a new node tends to link to the more connected nodes (“the richest get richer”) [41]. That means that for the CDs resulting in complex networks, a discovery or innovation may trigger new ones; new patents with similar content emerge and, eventually, generate a community. In other words, solubility enhancement was initially the ultimate goal of CD ICs. Later, novel effects based on improved solubility gave rise to cutting-edge consolidated and emergent applications.

### 3.2. Analysis of Patenting Trends by Text-Mining

The resulting networks indicate that most of the patents are related to solubility enhancement for the subsequent development of suitable dosage forms (i.e., solutions, powders, and tablets) for determined purposes, which could be somehow expected. However, knowing the patenting patterns over time will enrich the understanding of the CDs’ complex networks (see above, Section 3.1) and may give an insight into the possible technological opportunities in the field of CDs. Accordingly, we carried out further mining of the 1998 patent dataset, now guided by words corresponding to pharmaceutical dosage forms, aiming to inform the number of patents related to each of them and how they have evolved. We emphasized our analysis on solutions, powders, and tablets. However, we were motivated to mine other dosage forms to get a broader overview of the technological trends of CDs.

**Figure 6:**
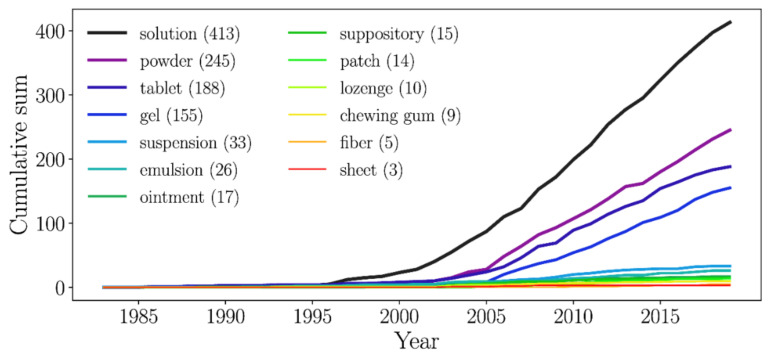
Patenting trends according to CD-based dosage forms.

Figure 6 shows that CDs are present in a diversity of dosage forms and most of the patents are related to aqueous solutions, tablets, and powders. The latter is in accordance with what network analysis pointed out (Section 3.1.5).

#### 3.2.1. Solutions

Most of the patents in our dataset refer to aqueous solutions, with 413 retrieved records (Figure 6). These outcomes support the observations arisen from Figure 4, showing that the network’s largest community is formed from patents related to aqueous solutions, certainly resulting from CD/drug-soluble ICs. This result is not surprising, as CDs have been primarily studied for their ability to form water-soluble ICs with poorly soluble compounds to yield aqueous solutions for parenteral, enteral, or local administration [91,92,93]. We highlight the case of ophthalmic and nasal solutions, described in the analysis of Community 11 (Section 3.1.5), which appeared as an emerging trend, as discussed in our previous work, which also mentions examples of patents issued for CD-based aqueous solutions for parenteral, nasal, and ocular administration [1]. 

Since the early 2000s, the number of issued patents in this area has grown significantly, and this trend remains the same up to this day, reflecting that the use of CDs for solubility enhancement is not old-fashioned. On the contrary, the modification of apparent solubility through CDs, although not a novelty, is still a very valid approach in pharmaceutics, including drug-repurposing and the development of innovative therapies, whose performance depends on the suitable solubility of one or more of their components. 

#### 3.2.2. Powders

Figure 6 shows that powders and tablets emerged jointly in the early 2000s, and, after 2005, both had substantial growth. However, it is notable that the number of patents for powders is higher since these patents may refer to powders as the final form (for example, for nasal or pulmonary administration) or to intermediates that are further reconstituted for parenteral administration or processed to produce other physical forms (i.e., tablets or hard gelatin capsules). These results are in accordance with Table 2, which shows the contribution of the word *powder* on defining communities in both *Novelty* and *Use* networks. 

An example of a powder technology as a final dosage form is a patent filed in 2019 by Pfizer Inc. (New York, NY, USA), comprising a tetrahydroquinazoline derivative, a KRAS inhibitor (one of the most hard-to-hit cancer-related proteins), in the form of dry powder for inhalation or intranasal administration; the formulation uses a CD as a solubilizer, stabilizer, taste-masking, and bioadhesive agent [94]. In another case, the βCD/ethinyl estradiol IC is patented as an amorphous powder for further processing into tablets, lozenges, or pellets. The complexation with βCD resulted in enhanced API solubility and stability and improved batch-to-batch reproducibility [95].

The patenting rate in CD-based powders is increasing, and thorough investigation into the role of CDs in the mucoadhesion, rheological and mechanical properties of powders may boost the development of CD-based powder technologies for innovative therapies, an already attractive emerging area.

#### 3.2.3. Tablets

Tablets are the most common dosage forms for oral administration. They are advantageous because of their high-precision dosing; chemical, physical, and microbiological stability; and affordable manufacturing processes. The latter generally comprises the compression of mixtures containing the active ingredient and one or more excipients. CDs play an essential role as multifunctional excipients for these dosage forms. CDs can work as direct compression fillers, binders, and disintegrating components of the mixture; they can stabilize the formulation and improve its organoleptic properties. Moreover, they can increase the dissolution rate, improving the oral bioavailability of the Biopharmaceutics Classification System Class II and IV drugs and modifying the API’s release profile. They can be used for uncoated and coated, orally disintegrating, effervescent, modified release, osmotic pump, chewable, mucoadhesive-buccal, and sublingual tablets [42].

All these recognized attributes give CDs a significant presence in the formulation of these dosage forms, as evidenced by the number of commercial tablets containing CDs (Table S1) and the significant increase in the number of patents since 2005 (Figure 6). Examples of patented technologies include a tablet containing an active compound to treat acute myeloid leukemia. In this formulation, HPβCD enables the required moldability, stability, ease of disintegration, and solubility, resulting in a composition of rapid dispersibility, dissolution, and bioavailability [96]. A βCD/progesterone IC, formulated as a rapidly disintegrating tablet for sublingual administration, demonstrating greater drug bioavailability, is another example of a recent patent [97]. Likewise, a newfangled innovative work claims that the use of insoluble and water-soluble fractions of a CD polymer conjugate generates a multifunctional excipient—with distinctive compression, drug dispersion, solubilization, and disintegration—for tablet formulations containing insoluble active ingredients [98].

Patents for tablets account for 188 files and are in the third place of importance after aqueous solutions and powders, also reflected in the communities formed through this word within the networks discussed before. Indeed, research around the multifunctional performance of CDs for oral, buccal, and sublingual tablets [99,100] would strengthen these technologies in the short term.

#### 3.2.4. Gels

Motivated by the course observed for patents related to the word *gel*, we included a brief discussion of it in this section, despite that *gel* was not in the 10 top-ranked words. Gels accounted for 155 records in the dataset. In 2010, their growth began to differ markedly, and numerous patents, reporting CDs to develop hydrogels for pharmaceutical applications, have been granted since then. 

In hydrogels, CDs can be covalently attached to give rise to supramolecular architectures with tunable drug release profiles. If CD cavities are available, they can host drug molecules while simultaneously preventing their release upon media dilution. Moreover, promising potential for delivering the drugs in a sequential manner or in response to a stimulus has been observed [101]. We briefly describe some examples of patents related to CD-based gels: a thermosensitive gel formed by polyethylene glycol, αCD, and polycaprolactone to deliver a neurolytic agent to treat certain cardiopulmonary conditions has been patented [102]; a thermoreversible system that gels upon contact with the body to sustain drug release, in which CDs act as temperature modulating agents (studies in guinea pigs, female sheep, rabbits, and Sprague–Dawley rats showed that the gel sustained the release of the active agent for periods of 5 to 7 days) [103]; an implant substance comprising an injectable tissue adhesive hydrogel, including γCD, used as a skin glue to promote tissue regeneration [104]; an aqueous system for local administration of antifungal or antipsoriatic drugs (i.e., hydrocortisone, triamcinolone acetonide, econazole) for treating nail diseases; it consists of a thermosensitive hydrogel, liquid at room temperature, that gels once applied on the nail’s surface, forming a thin film; a methylated derivative of βCD is used as a solubilizing agent, enabling high concentrations of hydrophobic active agents) [105].

The convergence of nanotechnology and pharmaceutical and materials sciences is reflected in nanosystems like the one patented by Fahmy et al. in 2017, which comprises a lipidic nanoshell surrounding a CD-based hydrogel containing a drug to treat the symptoms of inflammatory or autoimmune diseases [106].

Photo-responsive βCD supramolecular hydrogels for delivering drugs on demand [107], injectable αCD hydrogels for plasmid delivery [108], chitosan–βCD hydrogels for wound dressing [109], and gelatin–βCD self-healing gels for cell delivery [110] show how recent research is expanding the scope of fascinating CD-based hydrogel technologies. Hence, CD-based gels are an emerging technology, and a significant increase in research, patents, and innovations in this direction is expected in the years to come. 

#### 3.2.5. Other Dosage Forms

Figure 6 shows that the use of CDs for other dosage forms, namely, suspensions, emulsions, ointments, suppositories, patches, lozenges, chewing gums, fibers, and sheets, has been explored. The patenting rate has remained unchanged over time; nevertheless, CDs’ performance proves their versatility and potential as functional excipients for those formulations.

#### 3.2.6. Suspensions

In the case of suspensions, those for ophthalmic administration stand out, where it has been shown that CDs improve physical and chemical stability, can act as permeability enhancers, and decrease eye irritation [49,111,112]. For instance, there is a patent that describes an aqueous ophthalmic suspension comprising solid CD/drug (i.e., dexamethasone) particles, ranging from 10 nm to 1 mm in size, suspended in the aqueous phase to treat conditions of the posterior segment of the eye (impossible to reach with conventional eye drops), such as the vitreous and optic nerve. In vivo studies in female albino rabbits showed how the ocular suspension enhances drug delivery into the posterior segment of the eye [113].

CDs have also been helpful as suspending agents, thus enabling the development of suspensions intended for being administered by other routes. Such is the case of an invention registered in 2019 that presents the composition, methods, and systems for nasal or pulmonary delivery of a biologically active compound for the treatment of inflammatory or obstructive pulmonary disease through a metered-dose inhaler, where CDs control the solubility of the system and also act as suspending agents [114]. More investigation about CDs’ role in suspensions, their interaction with the rest of the formulation components, and their performance as suspending agents can expand their potential in these dosage forms.

#### 3.2.7. Emulsions

Emulsions can be prepared using CDs instead of surfactants [115] and are very useful in Pickering pharmaceutical emulsion stabilization [116]. In 2012, Laza-Knoerr et al. patented emulsions with remarkable stability, containing CD-based polymers and lipophilic compounds, the methods to obtain them, and their uses. Emulsion stabilization is given by host–guest interactions between the CD polymers and the lipophilic active ingredients without using organic solvents, surfactants, co-surfactants, or other additives, thus showing great potential for pharmaceutical applications [117]. Although CDs appear as an attractive tool to formulate surfactant-free emulsions, their application in these pharmaceutical technologies remains unrevealed.

#### 3.2.8. Chewing Gums

Medicated chewing gums represent a very particular dosage form useful for local (dental health or buccal therapies) or systemic drug delivery. For the latter, it overcomes the limitations associated with gastrointestinal drug degradation and first-pass metabolism. The drug release depends on its aqueous solubility: water-soluble drugs are fully and rapidly released, whereas poor-soluble substances are slowly and incompletely delivered [118]. CDs have been successfully used as solubilizers and taste-masking agents for these technologies [119]. There is an invention describing compressed chewing gums containing peptides of 5 to 11 amino acids (i.e., antimicrobial peptides) for their buccal administration. In these compositions, CD derivatives are used as absorption enhancers and bulk sweeteners [120]. Despite the challenges related to the formulation of medicated chewing gums, they represent a suitable alternative for drug administration for children, geriatrics, and patients with severe sore throat conditions. More investigation is required to expand the potential of these dosage forms. Lozenges, along with other formulations for oral and buccal administration, were discussed in our previous work [1].

#### 3.2.9. Patches and Fibers 

The transdermal patch has become an important pharmaceutical technology in the last decade due to its many advantages, such as the sustained delivery of drugs across the skin into systemic circulation while bypassing first-pass metabolism, avoidance of gastric drug degradation, and low dosage requirements. However, direct loading of low-soluble drugs into patches for their transdermal delivery is still challenging [121]. CDs have been demonstrated to overcome drug solubility limitations and improve stability when loaded into patches [122,123,124]. The same is true for buccal patches, in which the solubilizing and stabilizing abilities of CDs, combined with their mucoadhesive properties, clearly evidences the great potential of CDs for this aim [125]. CDs have also had a suitable performance in the fabrication of drug-loaded transdermal fibers [126] and even as wearable epidermal biosensors [127] and coated implants [128]. As expected, fascinating recent innovations have arisen in this field. One of them is a CD-collagen-based matrix composition used as a therapeutic eye patch for corneal repair. In this technology, CDs increased collagen thermal stability and reduced collagen fibrogenesis [129]. In 2015, an invention registered by Caltech described a layered polymeric monofilament fiber drug delivery device, suitable for implantation in a patient, to control the delivery of antibiotics or antimicrobial drugs to treat periodontitis and ocular diseases, among others. The device comprises at least two side-by-side layers exposed to the environment, and the drug release is tuned by controlling the characteristics of the individual fibers, such as chemical composition and structural design. Incorporating CDs through covalent or non-covalent interactions to the backbone polymer yields a structure (crosslinked through host–guest interactions) that enables the gradual release of the therapeutic agent over a determined period of time. Some of the performed in vitro studies showed that, in the implant, the two fibers sustained the release of levofloxacin over 10–15 days, while a third fiber released 90% of levofloxacin on the first day. In in vivo studies, in which the levofloxacin-loaded device was implanted in the eyes of New Zealand white rabbits, showed levels of levofloxacin, detected in tears, that were expected to have antimicrobial effects for 6 days [130].

Undoubtedly, CDs’ ability to modify the aqueous solubility of a guest molecule has positioned CDs in the pharmaceutical field and triggered novel new effects based or associated with it, making CDs applicable to the development of a variety of dosage forms. The progress in materials science, supramolecular chemistry, and nanotechnology may lead to striking innovations and, therefore, an increment in patenting of those dosage forms that have not received much attention but in which CDs could play a vital role. Patches and fibers must be underscored as they are backed up by recent fascinating research. The same is true for the suppositories discussed in our previous work [1]. The potential of CDs in suspensions and emulsions has yet to be recognized. Although the utility of CDs for chewing gums is undeniable, an in-depth investigation is needed for these alternative pharmaceutical forms.

## 4. Conclusions

Data science tools automatically enabled the immediate visualization of CD pharmaceutical technologies. CD patents formed complex networks that evidenced how solubility enhancement employing CDs has triggered cutting-edge applications for a variety of pharmaceutical purposes.

Most CD pharmaceutical patents were associated with the development of aqueous solutions for parenteral or local administration. Significant technological progress is observed for tablets, while gels seem to be very promising. Patches and fibers using CDs are emerging, and fascinating recent research backing them up may elicit an increment in their patenting trend in the short term. The potential of CDs in suspensions and emulsions has yet to be recognized, and a better understanding of CDs in these dosage forms will make these technologies a great opportunity area. Optimization of cancer therapies through CDs has been widely explored, while antiviral approaches could reach the same maturity level in the short term.

The interest in CDs is still increasing, and, based on our analysis, this trend will continue in the coming years due to their versatility and fascinating ability to improve pharmaceutical formulations in the design of innovative therapies. 

Through the approach presented herein, which can also be implemented to analyze scientific papers, we aim to contribute to the intercourse between scientific and technological literature that, in turn, in an integrated manner, can foster innovations for both academia and industry for CDs and other materials with potential in the pharmaceutical and drug delivery fields. 

## Figures and Tables

**Table 1:** CD-based formulation approvals in the last five years [6,7,8,9,10,11].

	Trade Name	Type of CD	API	Dosage Form/Administration Route	Company
2021	* Janssen COVID-19 Vaccine	HPβCD	Ad26.COV2-S	Suspension for I.M. administration	Janssen Biotech, Inc. (Horsham, PA, USA), a Janssen Pharmaceutical Company of Johnson & Johnson
2020	* Veklury	SBEβCD	Remdesivir	Lyophilized powder for I.V. solution	Gilead Sciences, Inc. (Foster City, CA, USA)
	** Trappsol Cyclo	HPβCD	Cyclodextrin	I.V. solution	Cyclo Therapeutics, Inc. (Gainesville, FL, USA)
2019	Zulresso	SBEβCD	Brexanolone	I.V. solution	Sage Therapeutics, Inc. (Cambridge, MA, USA)
	Baqsimi	βCD	Glucagon	Nasal powder	Eli Lilly and Company (Indianapolis, IN, USA)
2017	Voriconazole	HPβCD	Voriconazole	Lyophilized powder for I.V. solution	Xellia Pharmaceuticals ApS (Copenhagen, Denmark)

* Emergency use authorization by the FDA; ** FDA fast track process.

**Table 2:** Top 5 words found in network communities for (**a**) *Novelty* and (**b**) *Use*. C refers to community, W to word, and F to frequency.

**(a) *Novelty***	**C**	**Size**	**W I**	**F**	**W II**	**F**	**W III**	**F**	**W IV**	**F**	**W V**	**F**
	1	129	water	0.35	cyclodextrin	0.2	soluble	0.17	tablet	0.15	beta-cyclodextrin	0.13
	2	294	cyclodextrin	0.28	salt	0.25	pharmaceutical	0.21	component	0.14	active	0.11
	3	88	acid	0.63	cyclodextrin	0.14	beta-cyclodextrin	0.08	salt	0.08	solution	0.07
	4	112	solution	0.53	water	0.12	beta-cyclodextrin	0.12	aqueous	0.12	cyclodextrin	0.11
	5	102	sodium	0.33	cellulose	0.21	hydroxypropyl	0.16	beta-cyclodextrin	0.16	starch	0.15
	6	197	material	0.3	powder	0.28	beta-cyclodextrin	0.15	cyclodextrin	0.14	radix	0.14
	7	357	cyclodextrin	0.66	complex	0.17	water	0.08	solution	0.04	salt	0.04
	8	42	oil	0.41	mixture	0.18	volatile	0.17	beta-cyclodextrin	0.15	water	0.09
	9	302	beta-cyclodextrin	0.53	hydroxypropyl	0.23	acid	0.1	hydrochloride	0.07	cyclodextrin	0.07
**(b) *Use***	**C**	**Size**	**W I**	**F**	**W II**	**F**	**W III**	**F**	**W IV**	**F**	**W V**	**F**
	1	342	tablet	0.41	medicine	0.2	capsule	0.16	granule	0.13	medicinal	0.1
	2	59	sustained	0.58	hydrochloride	0.25	antibacterial	0.06	patient	0.05	drug	0.05
	3	136	cancer	0.64	cell	0.13	disease	0.08	breast	0.08	lung	0.07
	4	175	drug	0.62	tumor	0.11	anti-inflammatory	0.11	carrier	0.09	treat	0.08
	5	238	disease	0.55	disorder	0.23	syndrome	0.11	inflammatory	0.06	chronic	0.05
	6	183	pharmaceutical	0.57	drink	0.14	animal	0.11	cosmetic	0.09	drug	0.09
	7	256	pain	0.39	complex	0.22	bone	0.14	cyclodextrin	0.13	oral	0.12
	8	16	particle	0.58	peptide	0.19	active	0.08	ingredient	0.08	heat	0.08
	9	124	injection	0.42	powder	0.24	freeze	0.14	dried	0.14	soluble	0.05
	10	151	treatment	0.32	infection	0.31	virus	0.18	disease	0.13	medicament	0.07
	11	99	eye	0.28	nasal	0.28	allergic	0.18	drop	0.14	macular	0.12

## Data Availability

The codes are available from the authors upon request.

## Supplementary Materials

The following are available online at https://www.mdpi.com/article/10.3390/pharmaceutics13081297/s1, Table S1: Commercially available CD-based formulations. References [6,7,8,9,10,11,42,90,131,132,133] are cited in the supplementary materials.
