# Supplementary material for: When Cyclodextrins Met Data Science: Unveiling Their Pharmaceutical Applications through Network Science and Text-Mining
Source: Pharmaceutics. 2021 Aug 19;13(8):1297. doi: 10.3390/pharmaceutics13081297 (PMC8399453; doi:10.3390/pharmaceutics13081297)
Supplement: Supplementary file 1 [file pharmaceutics-13-01297-s001.zip › pharmaceutics-1326409-supplementary.pdf]

pharmaceutics

Supplementary Material: When Cyclodextrins Met Data Science: Unveiling Their Pharmaceutical Applications through Network Science and Text-Mining

Juliana Rincón-López, Yara C. Almanza-Arjona, Alejandro P. Riascos and Yareli Rojas-Aguirre

Table S1. Commercially available CD-based formulations [1–11].

Trade name	Type of CD	API	Administration route/dosage form	Indication	Company
**Janssen COVID-19 Vaccine**	HPβCD	Ad26.COV2-S	Suspension for I.M. administration	Vaccine (COVID-19)	Janssen Biotech, Inc. (Horsham, Pennsylvania, USA)
**Veklury**	SBEβCD	Remdesivir	Lyophilized powder for I.V. solution	Antiviral (COVID-19)	Gilead Sciences, Inc. (Foster City, California, USA)
**Trappsol Cyclo**	HPβCD	Cyclodextrin	I.V. solution	Niemann-Pick Disease Type C	Cyclo Therapeutics, Inc. (Gainesville, Florida, USA)
**Zulresso**	SBEβCD	Brexanolone	I.V. solution	Postpartum depression	Sage Therapeutics, Inc. (Cambridge, Massachusetts, USA)
**Baqsimi**	βCD	Glucagon	Nasal powder	Severe hypoglycemia	Eli Lilly and Company (Indianapolis, Indiana, USA)
**Voriconazole**	HPβCD	Voriconazole	Lyophilized powder for I.V. solution	Broad spectrum antimycotic	Xellia Pharmaceuticals ApS (Copenhagen, Denmark)
**Pazeo**	HPγCD	Olopatadine hydrochloride	Ocular solution	Allergic conjunctivitis	Alcon Research Ltd. (Geneva, Switzerland)
**Pansporin T**	αCD	Cefotiam hexetil hydrochloride	Oral tablet	Antibiotic	Takeda Pharmaceutical Co. (Osaka, Japan)
**Brexin**	βCD	Piroxicam	Oral tablet	Analgesic and anti-inflammatory	Chiesi Pharmaceuticals (Parma, Italy)
**Opalmon**	γCD	Limaprost	Oral tablet	Burger's disease	Ono Pharmaceutical Co., Ltd. (Osaka, Japan)
**Meiact**	βCD	Cephalosporin	Oral tablet	Antibiotic	Meiji Seika Pharma Co., Ltd. (Tokyo, Japan)
**Glymesason**	βCD	Dexamethasone	Oral tablet, ointment	Analgesic and anti-inflammatory	Fujinaga Pharmaceutical Co., Ltd. (Tokyo, Japan)
**Yaz**	βCD	Ethinylestradiol and drospirenone	Oral tablet	Contraception	Bayer AG (Leverkusen, Germany)
**Mobitil**	βCD	Meloxicam	Oral tablet, rectal suppository	Non steroid anti-inflammatory	Medical Union Pharmaceuticals (Cairo, Egypt)
**Surgamyl**	βCD	Tiaprofenic acid	Oral tablet	Analgesic	Roussel-Maestrelli (Paris, France)
**Cicladol**	βCD	Piroxicam	Oral tablet, rectal suppository	Anti-inflammatory	Masterpharma (Parma, Italy)
**Lonmiel**	βCD	Benexate hydrochloride	Oral capsule	Gastric ulcer	Negase Medicals Co. (Itami, Japan)
**Omebeta**	βCD	Omeprazole	Oral tablet	Gastric ulcer	Betapharm (Augsburg, Germany)
**Zyrtec**	βCD	Cetirizine	Chewable tablet	Respiratory allergies	Losan Pharma GmbH (Neuenburg am Rhein, Germany)

**Stada-Travel**	βCD	Diphenhydramine and chlorotheophylline	Chewable tablet	Travel sickness	Stada Arzneimittel AG (Bad Vilbel, Germany)
**Nicorette**	βCD	Nicotine	Sublingual tablet	Abstinence syndrome	Pharmacia Upjohn (Now owned by Pfizer Inc., NYC, New York, USA)
**Nitropen**	βCD	Nitroglycerin	Sublingual tablet	Coronary dilatator	Nippon Kayaku Co. (Tokyo, Japan)
**Prostarmon E**	βCD	PGE_2	Sublingual tablet	Labor induction	Ono Pharmaceutical Co., Ltd. (Osaka, Japan)
**Mena-Gargle**	βCD	Iodine	Oral solution (Gargling)	Throat disinfection	Kyushin pharmaceutical Co., Ltd. (Tokyo, Japan)
**Sporanox**	HPβCD	Itraconazole	Oral solution	Antimycotic	Janssen Pharmaceutica (Beerse, Belgium)
**Flogene**	βCD	Piroxicam	Oral solution	Analgesic and anti-inflammatory	Ache Laboratorios Farmaceuticos S.A. (São Paulo, Brasil)
**Nimedex**	βCD	Nimesulide	Oral sachet	Non steroid anti-inflammatory	Novartis International AG (Basel, Switzerland)
**VFEND**	SBEβCD	Voriconazole	I.V. solution	Antimycotic	Pfizer, Inc. (NYC, New York, USA)
**Cardiotec**	HPγCD	Tc-99 and Teboroxime	I.V. solution	Radioactive imaging	Bracco Group (Milan, Italy)
**Caverject Dual**	αCD	Alprostadil	Intracarvernosal injection	Erectile disfunction	Pfizer, Inc. (NYC, New York, USA)
**MitoExtra**	HPβCD	Mitomycin	I.V. solution	Cancer	Novartis International AG (Basel, Switzerland)
**Prostavasin**	αCD	PGE_1	Intra-arterial or I.V. solution	Chronic arterial occlusive disease	Ono Pharmaceutical Co., Ltd. (Osaka, Japan)
**Edex**	αCD	PGE_1	Solution for Intracavernosal injection	Erectile disfunction	Schwarz PharmaAG (Acquired by UCB pharma, Brussels, Belgium)
**Zeldox, Geodon**	SBEβCD	Ziprasidone	Oral capsule, I.M. solution	Schizophrenia	Pfizer, Inc. (NYC ,New York, USA)
**Lubion**	HPβCD	Progesterone	I.M. solution or solution for subcutaneous injection	Infertility treatment	Hikma Pharmaceuticals (London, UK)
**Indocid**	HPβCD	Indomethacin	Ocular solution	Ocular inflammation and cystoid macular edema	Chauvin Pharmaceuticals Ltd. (London, UK)
**Clorocil**	RMβCD	Chloramphenicol	Ocular solution	Antibiotic	Oftalder (Lisboa, Portugal)
**Voltaren Optha**	HPγCD	Diclofenac sodium salt	Ocular solution	Non steroid anti-inflammatory	Novartis International AG (Basel, Switzerland)
**Aerodiol**	RMβCD	17β-Estradiol	Nasal spray	Hormone therapy	Servier Laboratories (Suresnes, France)
**Coordinax**	βCD	Cisapride	Rectal suppository	Gastrointestinal mobility stimulant	Janssen Pharmaceutica (Beerse, Belgium)
**Acerap**	βCD	Aceclofenac	Tablet	Osteoarthritis, muscular pain, gout, rheumatoid arthritis	Taj Pharma India (Mumbai, India)
**Betahist**	βCD	Betahistine	Tablet	Vertigo Ménière's syndrome	Geno Pharmaceuticals Pvt. Ltd. (Mapusa, India)
**Transilium**	βCD	Chlordiazepoxide	Tablet	Anxiety, neurosis, psychosis	Gador (Buenos aires, Argentina)
**Cold Remedy Soothing**	βCD	Chlorpheniramine maleate, acetaminophen	Tablet	Fever, allergies	Foshan Dezhong Pharmaceutical Co. Ltd. (Guangdong, China)

**Natures Aid Vitamin D3**	βCD	Cholecalciferol	Tablet	Vitamin D deficiency	Natures Aid (Preston, UK)
**Rynathisol**	βCD	Dextromethorphan	Tablet	Cough	Synthelabo Pharmaceuticals (Now Sanofi S.A., Paris, France)
**Fluner**	βCD	Flunarizine	Tablet	Migraine occlusive peripheral vascular disease	Geno Pharmaceuticals Pvt. Ltd. (Mapusa, India)
**Entronor-TZ**	βCD	Norfloxacin, tinidazole	Tablet	diarrhea, protozoal infections	Sydler (Mumbai, India)
**Cycladol**	βCD	Piroxicam	Tablet, suppository, oral powder	Osteoarthrosis, rheumatoid arthritis, ankylosing, spondylitis	Chiesi Pharmaceuticals (Parma, Italy)
**Rofizgel**	βCD	Rofecoxib	Tablet	Osteoarthrosis, rheumatoid arthritis, ankylosing, spondylitis	Wockhardt Ltd. (Mumbai, India)
**Perindopril Erbumine**	HPβCD	Perindopril tert-butylamine	Tablet	Hypertension, heart failure and cardiac events prevention	Sandoz Pharmaceuticals S.A. (Part of Novartis International AG, Basel Switzerland)

References

1. Szejtli, J. Past, Present, and Future of Cyclodextrin Research. *Pure Appl. Chem.* **2004**, *76*, 1825–1845, doi:10.1351/pac200476101825.
2. Loftsson, T.; Brewster, M.E. Pharmaceutical Applications of Cyclodextrins: Basic Science and Product Development. *J. Pharm. Pharmacol.* **2010**, *62*, 1607–1621, doi:10.1111/j.2042-7158.2010.01030.x.
3. Conceição, J.; Adeoye, O.; Cabral-marques, H.M.; Manuel, J.; Lobo, S. Cyclodextrins as Excipients in Tablet Formulations. *Drug Discov. Today* **2018**, *23*, 1274–1284, doi:10.1016/j.drudis.2018.04.009.
4. Fenyvesi, É.; Puskás, I.; Szente, L. Applications of Steroid Drugs Entrapped in Cyclodextrins. *Environ. Chem. Lett.* **2019**, *17*, 375–391, doi:10.1007/s10311-018-0807-7.
5. Sohajda, T. Cyclo Therapeutics Announces Design of Pivotal Phase 3 Study Evaluating Trappsol® Cyclo™ in Niemann-Pick Type C1 Available online: https://cyclodextrinnews.com/2021/04/29/cyclo-therapeutics-announces-design-of-pivotal-phase-3-study-evaluating-trappsol-cyclo-in-niemann-pick-type-c1/ (accessed on 7 July 2021).
6. FDA Pazeo (Olopatadine Hydrochloride) Ophthalmic Solution FDA Approval Available online: https://www.accessdata.fda.gov/drugsatfda_docs/nda/2015/206276Orig1s000TOC.cfm (accessed on 12 July 2021).
7. FDA Drug Approval Package: Voriconazole for Injection Available online: https://www.accessdata.fda.gov/drugsatfda_docs/nda/2017/208562Orig1s000TOC.cfm (accessed on 12 July 2021).
8. FDA FDA-Approved Drugs: Baqsimi Available online: https://www.accessdata.fda.gov/scripts/cder/daf/index.cfm?event=overview.process&ApplNo=210134 (accessed on 12 July 2021).
9. FDA Drug Approval Package: Zulresso Available online: https://www.accessdata.fda.gov/drugsatfda_docs/nda/2019/211371Orig1s000TOC.cfm (accessed on 12 July 2021).
10. FDA COVID-19 Update: FDA Broadens Emergency Use Authorization for Veklury (Remdesivir) to Include All Hospitalized Patients for Treatment of COVID-19 Available online: https://www.fda.gov/news-events/press-announcements/covid-19-update-fda-broadens-emergency-use-authorization-veklury-remdesivir-include-all-hospitalized (accessed on 6 July 2021).
11. FDA Janssen COVID-19 Vaccine | FDA Emergency Use Authorization Available online: https://www.fda.gov/emergency-preparedness-and-response/coronavirus-disease-2019-covid-19/janssen-covid-19-vaccine#additional (accessed on 6 July 2021).
